# Genome Assembly Has a Major Impact on Gene Content: A Comparison of Annotation in Two *Bos Taurus* Assemblies

**DOI:** 10.1371/journal.pone.0021400

**Published:** 2011-06-22

**Authors:** Liliana Florea, Alexander Souvorov, Theodore S. Kalbfleisch, Steven L. Salzberg

**Affiliations:** 1 Center for Bioinformatics and Computational Biology, University of Maryland, College Park, Maryland, United States of America; 2 National Center for Biotechnology Information, National Institutes of Health, Bethesda, Maryland, United States of America; 3 Center for Genetics and Molecular Medicine, University of Louisville, Louisville, Kentucky, United States of America; The University of Maryland, United States of America

## Abstract

Gene and SNP annotation are among the first and most important steps in analyzing a genome. As the number of sequenced genomes continues to grow, a key question is: how does the quality of the assembled sequence affect the annotations? We compared the gene and SNP annotations for two different *Bos taurus* genome assemblies built from the same data but with significant improvements in the later assembly. The same annotation software was used for annotating both sequences. While some annotation differences are expected even between high-quality assemblies such as these, we found that a staggering 40% of the genes (>9,500) varied significantly between assemblies, due in part to the availability of new gene evidence but primarily to genome mis-assembly events and local sequence variations. For instance, although the later assembly is generally superior, 660 protein coding genes in the earlier assembly are entirely missing from the later genome's annotation, and approximately 3,600 (15%) of the genes have complex structural differences between the two assemblies. In addition, 12–20% of the predicted proteins in both assemblies have relatively large sequence differences when compared to their RefSeq models, and 6–15% of bovine dbSNP records are unrecoverable in the two assemblies. Our findings highlight the consequences of genome assembly quality on gene and SNP annotation and argue for continued improvements in any draft genome sequence. We also found that tracking a gene between different assemblies of the same genome is surprisingly difficult, due to the numerous changes, both small and large, that occur in some genes. As a side benefit, our analyses helped us identify many specific loci for improvement in the *Bos taurus* genome assembly.

## Introduction

Recent years have seen a tremendous increase in the number and diversity of sequenced genomes. More than 350 eukaryotes have been sequenced and 400 more are planned (http://www.ncbi.nlm.nih.gov/genomes/leuks.cgi). Most model organisms so far have been sequenced under the umbrella of large genome projects undertaken by large international consortia. A typical genome project produces a first draft of the genome, together with annotations and analyses. Subsequent releases then correct or alleviate assembly problems and update the auxiliary information. Thus, a genome project traditionally sets up a continuous effort to update these resources. New sequencing technologies have dramatically accelerated the pace at which new genomic sequences are being produced; they can now produce in less than one week the amount of data originally generated to sequence the human genome. The short reads produced by new sequencers, although a major challenge to assembly algorithms, have already become the standard due to their dramatically lower cost. Several large genomes have already been assembled from short reads or from a combination of short and traditional Sanger reads [1]–[5]. Low cost, large sequencing capacity along with increasingly better assembly algorithms will soon make it possible for smaller groups, even individual investigators, to sequence and assemble their organisms of interest. Virtually all of these projects will produce “draft” genomes, in which the chromosomes are assembled into a relatively large number of contiguous fragments (contigs) separated by gaps, and annotation software will then use these contigs, typically within groups of contigs with known order and gap sizes (scaffolds) or full chromosomes, as the substrate on which to identify protein-coding genes.

Once a draft genome sequence is produced, the first and most crucial step in its analysis is finding the genes. Knowing the correct location and structure of a gene provides the basis for downstream studies of gene function. Gene annotation remains a difficult problem as reflected by the fact that, ten years after sequencing the human genome, there is still no consensus on the number and structure of human genes [6]–[10]. A significant complication arises when errors in the assembly interfere with the correct annotation, such as by deleting or scrambling the order of exons, or by altering a gene's sequence. (Note that unless otherwise specified, we use “genes” to refer only to protein-coding genes.) Indeed, even minute sequence changes such as frameshifts or nucleotide substitutions can dramatically modify the predicted protein. Here we focus on how assembly quality affects gene annotation, and how this in turn appears to scientists using the annotation.

One important genome analysis that is quickly available after the sequencing of a new diploid genome is a catalog of sequence variations, in particular single nucleotide polymorphisms (SNPs). The human HapMap project [11] was the first effort of scientists and organizations worldwide to collect variations in the genome sequences of individual humans. These freely available data allow scientists to search for common patterns of variation or for patterns associated with specific conditions. This collaborative model has since been adopted, albeit at a smaller scale, by many other genome sequencing projects. For instance, more than two million cattle SNPs have been collected in the dbSNP repository in GenBank, and the Bovine HapMap has recently reported a genome-wide characterization of >37,000 SNPs, which in turn has revealed patterns of variation associated with cattle domestication, selection and breed formation [12]. Regardless of the species analyzed, collecting such information requires a tremendous effort that cannot be easily replicated. Mapping SNP information reliably onto a new version of the genome is crucial to preserving these efforts, but errors in the assembly can interfere. In our analysis below, we look at how the *Bos taurus* assembly affects the ability to recover the SNP information.

Our group has assembled and released two successive versions of the cow genome (UMD2 and UMD3; [13]). Both assemblies were produced with the open source Celera Assembler software (https://sourceforge.net/projects/wgs-assembler/), modified and augmented with additional algorithms from our group, and both assemblies used the identical input (Sanger) sequences from the NCBI Trace Archive. UMD2 contains 2.61 billion bases (Gbp) in 30 chromosomes (1–29 and X) and 240 Mbp of unplaced sequence. UMD3, which was built using an improved algorithm and the identical input data, contains 2.64 Gbp in 30 chromosomes and 9 Mbp of unplaced sequence. Both assemblies underwent extensive post-processing to maximize the amount of sequence that was mapped to the chromosomes. Additional assembly steps improved the order and orientation of contigs using paired-end sequence information, marker mapping, and synteny with the human genome. By all measures, UMD3 is a higher quality assembly than UMD2, with fewer gaps, smaller gaps, longer contigs and scaffolds, and more chromosomal sequence (**Table 1**). A major factor contributing to the improvements in UMD3 was more thorough filtering of contaminated reads and trimming of vector sequences, which allowed more overlapping reads to be assembled into contigs and more contigs into scaffolds, and which eliminated false joins that introduced errors in the assembly's order and orientation. The two genomes were deposited in GenBank upon release, and were annotated *de novo* using the NCBI eukaryotic genome annotation pipeline. The annotation procedure was run from scratch for the later assembly, as opposed to projecting original gene coordinates onto the new sequence, thus producing an unbiased annotation.

**Table 1:** Assembly statistics for UMD2 and UMD3.

Measure	UMD2	UMD3
Total sequence	2.850 Gbp	2.649 Gbp
In chromosomes (placed)	2.610 Gbp (91%)	2.640 Gbp (99%)
Unplaced	240 Mbp	9 Mbp
Number of contigs	75,775	70,770
Contig N50	88,288 bp	103,785 bp
Number of scaffolds	134,667	39,978
Scaffold N50	7.9 Mp	8.2 Mbp
Number of gaps	59,983	50,156
Gap N50	60,968 bp	34,758 bp

The N50 statistic is the minimum length of a feature (contig, scaffold, gap) such that using equal or longer features produces half of the bases of the genome-wide total.

These two genomes and their annotations represent the first time that two versions of a mammalian genome, based on the same raw data and differing only in the assembly methods used, have been annotated separately using the same method. This gives us a unique opportunity to catalog and quantify the effects of genome assembly on gene annotation and, by implication, on downstream analyses.

In comparing the gene and SNP annotations between UMD2 and UMD3, we considered these questions. First, how do changes in the assembly affect the *structure* of genes? Second, what effects do assembly errors have on the predicted protein sequences? And third, how does assembly quality affect our ability to detect SNPs? We compared the gene structures to determine commonalities and differences between transcripts from the two annotations, and then used sequence comparisons to quantify the effects of assembly errors on the predicted proteins. Separately, we compared the mapping rates of more than two million SNPs onto the two assemblies as a measure of assembly completeness and reliability.

Even though the two assemblies are highly similar, we found significant differences in gene content and gene structures, making it difficult to track a particular gene across multiple assemblies. There were also significant differences in the SNPs that could be mapped unambiguously. Further, as we show below, many assembly errors are directly reflected in the annotation, arguing for the need to continuously improve the sequence beyond the first assembly. As an added benefit, these analyses have helped identify specific loci in the *Bos taurus* genome sequence that can be improved for the benefit of its users.

## Results

We compared the *de novo* gene annotations between the two *Bos taurus* assemblies, to identify both common and assembly-specific genes and to quantify finer-grained differences in gene structure. Since we do not differentiate between transcripts and protein-coding genes in our analyses, we will be using the terms interchangeably. We separately assessed the impact of assembly quality on the predicted proteins, using a control set of known and reliable protein models. We also evaluated the ability to reliably map SNPs onto a target unfinished genome, for later use in population studies and in genotype-phenotype analyses.

### Comparison of gene annotations

Changes in the content, order and orientation of contigs in an assembly will necessarily bring about changes in its annotation, the nature and extent of which cannot be readily estimated. New transcripts might appear, others can be truncated, extended or shuffled, and some might disappear entirely. To assess the nature and extent of such differences, we first compared the gene content of the two assemblies, mapping each gene set to the other genome's sequence. We then compared transcripts between the two annotation sets based on their exon-intron structure. A secondary but important goal was to develop methods and tools to address the question: given a gene, how has it changed between the two assemblies? Answering this question is critical for researchers attempting to track genes from one version of a genome to the next.

Although most genes were present in both assemblies, the direct comparison of the two sets of annotations revealed unexpectedly complex results. At the surface, we found many named genes (characterized based on close sequence homology to known genes) appearing in both annotations, but we also found a large number of new and uncharacterized loci unique to each set. The latter could largely be attributed to the different evidence used by the annotation pipeline for the two annotations, a pattern that is likely to characterize organisms for which the cDNA and protein resources are constantly changing. Upon closer inspection, even in cases where the gene name was preserved, the gene structure often changed substantially. In fact, less than two thirds of the predicted genes in UMD2 (13,854 out of 23,221) have preserved their exon-intron structure in UMD3. Below we describe the nature of the differences we encountered, and the likely role assembly inaccuracies played in shaping them.

#### Gene content of the two genomes

When comparing the gene content between the two assemblies, most annotated transcripts in one assembly had at least partial sequence matches on the other assembly. However, for many transcripts there were significant differences in organization. For consistency, we will describe the comparison primarily from the perspective of mapping the UMD2 annotation onto the UMD3 assembly, because the latter is a more recent and higher-quality version of the *Bos taurus* genome.

**Figure 1:**
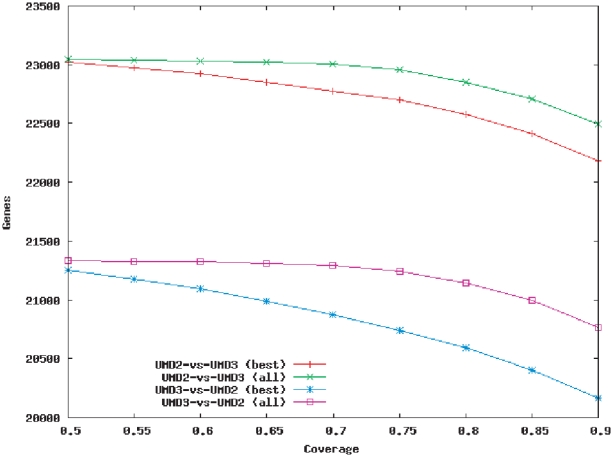
Mapping rates of annotated gene sequences between the UMD2 and UMD3 assemblies. Plotted values represent the numbers of genes in one annotation that have coverage *x* or larger in the other genome, where coverage refers to the proportion of the transcript covered. All: all alignments of a transcript are used to compute coverage; best: only the best alignment is used. For example, the red line shows that just over 23,000 genes from UMD2 have at least 50% of their sequence (coverage 0.5) aligned to a corresponding gene in UMD3. The total number of annotated protein-coding genes is 23,221 for UMD2, and 21,342 for UMD3.

As expected, only a relatively small number (160) of transcripts were missing from the newer UMD3 assembly. Most of these code for hypothetical proteins, and Blast [14] searches showed them to be of bacterial origin, representing contaminants in the original sequence data. They were therefore correctly excluded from the newer assembly. Only two UMD2 genes, *CEBPB* and *RNH1*, represented known genes, and *RNH1* had a short partial match on the UMD3 assembly that could not be detected with the search parameters. (Conversely, two UMD3 transcripts were missing from UMD2, both of them annotated as partial and lacking functional assignment.) In contrast, a relatively large number of transcripts were fragmented or incomplete, as shown in **Figure 1**. There were 878 transcripts from UMD2 for which the primary alignment on the genome contained less than 90% of the gene. Although this number improved when secondary alignments were included, 567 transcripts could still not be fully accounted for. Thus, while each assembly contains at least parts of nearly every transcript in the other genome, inconsistencies between the assemblies cause significant fragmentation of many genes.

A simple example is that of the *IL22* gene. The gene is complete in UMD2, with 6 exons between positions 45,880,245 and 45,886,101 on chromosome 5, derived from the spliced alignment of a RefSeq mRNA (accession NM_001098379). In contrast, the UMD3 version of the gene (positions 45,721,767–45,727,535 on the same chromosome) is missing a 200 bp segment from the core of exon 6 (positions 679–869 in the RefSeq mRNA), which falls within a gap in the assembly. A more complex example is the *SPOCK1* gene, which is complete in UMD3 but fragmented in UMD2. *SPOCK1* gene annotations were derived from the RefSeq mRNA NM_001075500. In UMD3, all 12 exons of *SPOCK1* are present, spaced over a 760 Kb region (bases 48,450,300–49,210,213 on the reverse strand of chromosome 7). In contrast, the alignment of the gene on UMD2 shows that exon 7 is missing. A secondary alignment finds the 115-bp exon 7 in the long (301 kbp) intron between exons 3 and 4 (bases 49,071,505–49,071,619). We traced the fragmentation of this gene to an incorrectly translocated contig in UMD2.

#### Gene structure comparison between the two assemblies

To assess the differences at a finer-grained level and to determine matching transcripts between the two annotation sets, we compared the exon-intron structures of genes, first mapping one set onto the other assembly with a spliced alignment method, and then comparing the coordinates against the local annotation (see **Methods**).

**Figure 2:**
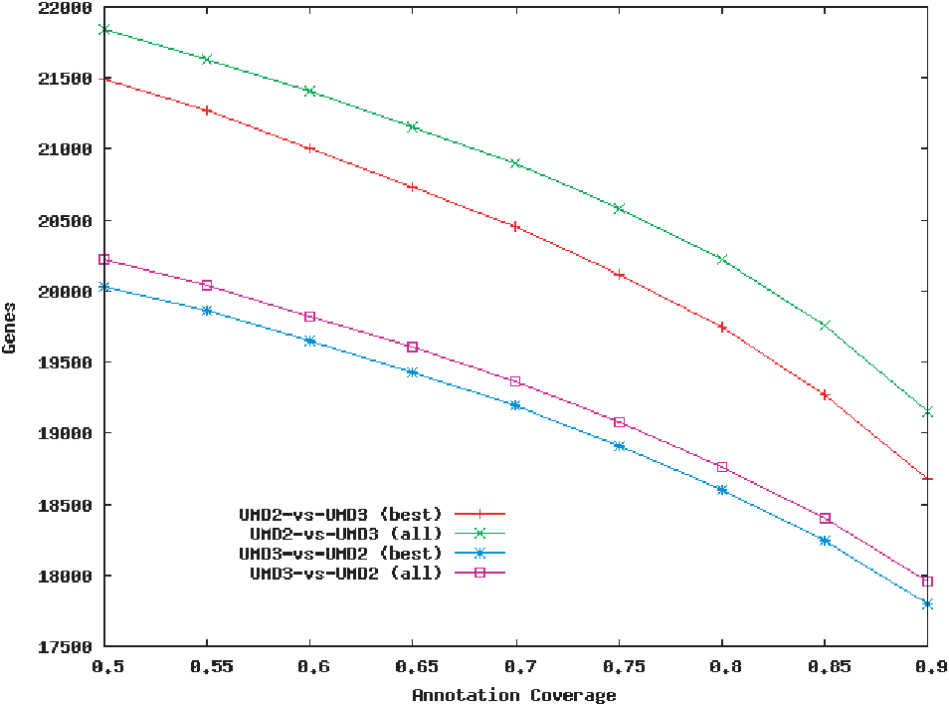
Agreement between gene annotations in the UMD2 and UMD3 assemblies. Plotted values represent the numbers of genes in one annotation that overlap the other annotation by a fraction *x* or more of their length, when all (‘all’) and when only the best (‘best’) alignments of a transcript are included. The total number of annotated protein-coding genes is 23,221 for UMD2, and 21,342 for UMD3.

When searching for a gene in a new genome, one is tempted to select the best hit, which is how many annotation systems operate. In our case, using only the best hit would leave 908 genes in UMD2 that had no overlap at all with the UMD3 annotation, and many genes that overlapped only partially. This unexpected result occurs because some of the annotated genes contain only a partial transcript due to inversions, translocations, or deletions in one or both assemblies, as we will illustrate with examples below. (**Figure 2** shows the fraction of each gene that overlapped with, or was ‘covered’ by, the local annotation.) Most of these genes were uncharacterized loci, and only 69 were named genes.

**Figure 3:**
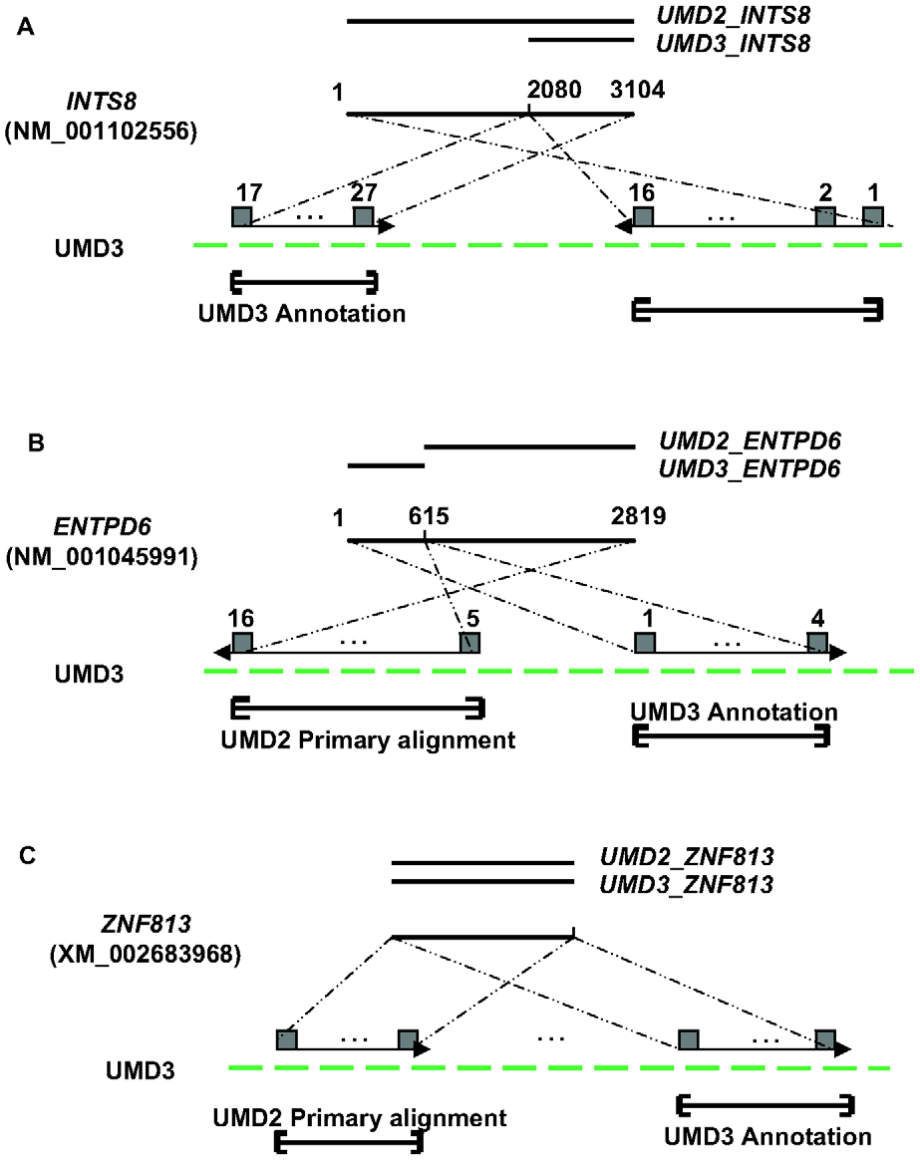
Examples showing how the same gene annotated on two assemblies completely fails to overlap. A) The alignment of the RefSeq DNA sequence for *INTS8* spans the entire gene on UMD2, but is truncated on UMD3. The figure shows how the *INTS8* sequence aligns to two distinct locations on UMD3, a longer, primary alignment containing exons 1–16 and a shorter one containing exons 17–27. The annotation system chose the shorter alignment (on the left) for the UMD3 annotation, which is thus disjoint from the primary alignment of the UMD2 annotation of *INTS8.* B) The gene *ENTPD6* is fragmented in both assemblies, and different segments were used by the annotation software in each case. Again, the primary alignment on UMD3 and the local annotation are distinct. C) The gene *ZNF813* has multiple matches on UMD3, but the best match and the annotated gene are disjoint.

The reasons why these genes were missing their potential targets turned out to be diverse (**Figure 3**). For example, in the case of *INTS8* the RefSeq model (NM_001102556) has two alignments on opposite strands of chromosome 14. The two alignments (exons 1–16, 1002 bp; and exons 17–27, 2080 bp) cover different portions of the gene and are inverted in UMD3 due to a contig rearrangement. While the primary alignment does not match any of the UMD3 annotations, the secondary, shorter alignment is co-located with the annotation of *INTS8*. Another example is *ZNF813*, which has several complete matches on UMD3, but the best alignment (positions 58,764,081–58,766,228 on chromosome 18, at 99% identity) and the annotated *ZNF813* (positions 60,286,463–60,305,339) do not coincide. In a third case, the two annotations contain complementary parts of the *ENTPD6* gene. Here, errors in both assemblies caused fragmentation of the RefSeq alignments, with two disjoint segments being included in the two annotation sets. Therefore, in many cases, even though the primary alignment does not lead to the desired gene, a secondary alignment does so. In fact, we recovered almost a third of the missing genes when we included secondary alignments, and the overall concordance between the UMD2 and UMD3 annotation was also improved (**Figure 2**). UMD2 still contained a significant number of truly unique genes (660), including 50 named genes, such as *SOX11*, *BAZ1A*, *FLNC*, *BrunoL5*, etc. We hypothesize that most of these resulted from the evolving cDNA and protein evidence available when annotating the two genomes rather than from changes in the structure of the assembly. Indeed, only 18 of these had potential matches among the UMD3 gene sequences, with only one of the alignments (for a 96 bp gene) complete.

Even though more than 95% of the genes are shared at least partially between the two annotation sets, specifying a one-to-one relationship is nearly impossible. Only 73.4% (17,052) of the UMD2 transcripts have a clear one-to-one correspondence to a transcript in the UMD3 annotation, and that number is reduced to 15,024 (65%) when all alignments are used. Gene fragmentation and paralogy within gene families are the primary reasons why one-to-many or many-to-many matches occur, but different numbers of splice sites, merged genes, and fused genes also account for some of the ambiguity. Moreover, only 13,854 (59.7%) have best matches with identical exon-intron structure in the UMD3 annotation. The rest of the genes with matches in the UMD3 annotation have an extension (14%), a truncation (6%), or a more complex rearrangement (16%) among the UMD3 predictions (**Table 2**). Such complex rearrangements, found in roughly 3600 genes, appear as missing or additional internal exons, different exon boundaries, or different combinations of exons.

**Table 2:** Best matches for UMD2 transcripts in the UMD3 annotation determined based on compatibility of exon-intron structures.

Classification	# Transcripts
Identical	13,854 (62.2%)
Extensions	3,157 (14.2%)
Truncations	1,329 (6.0%)
Both extensions & truncations	309 (1.4%)
Complex structural differences	3,607 (16.2%)
**Total**	**22,256**

Comparisons include a margin V = 20 of error at exon and intron boundaries.

As these results indicate, tracking genes between different assemblies and annotations is not easy, even for very similar genomes such as UMD2 and UMD3, which are based on identical underlying sequence data. Difficulties arise not only from assembly errors that alter the true structures of genes, but also from the evolving gene and protein evidence used by the gene annotation tool. From a user perspective, one immediate consequence is that changes in the assembly often make it difficult to transfer genes and their surrounding context information between different versions of a genome.

While comparative analyses such as these can pinpoint differences between assemblies, they cannot always help resolve the differences between assemblies, or even determine which gene models are correct. Next we look at how the accuracy of the assembly is directly reflected in the annotation quality.

### Effects of genome quality on protein annotation

Perhaps the most compelling way to look at how even small errors in the assembly affect the quality of its annotation is by analyzing their effects on the predicted proteins. The NCBI annotation system, which employs a conservative evidence-based process, reports a model RefSeq protein for each predicted protein. We were therefore able to compare the predicted proteins' conceptual translations (*i.e.*, direct end-to-end translation from the genomic annotation, prior to review and curation) to their validated RefSeq models to characterize differences, and thus to establish unequivocally the impact of assembly errors on protein integrity.

For each annotated protein-coding gene in each assembly, we compared protein sequences and classified their differences (**Table 3**). For an objective measure of accuracy, we only used pairs with curated RefSeq models (8,867 for UMD2 and 8,659 for UMD3). A majority of proteins in each genome were identical or near-identical with their RefSeq models (81% for UMD2; 89% for UMD3), and a small number (5% for UMD2 and 2% for UMD3) were near-identical but were either longer (extensions) or shorter (truncations) than the RefSeq models. The remaining 14% (UMD2) and 9% (UMD3) exhibited a wide variety of differences from RefSeq, including large gaps, internal divergent sequence or divergent sequence ends, and other more complex differences. As these results indicate, UMD3 looked consistently better than UMD2, with more similar sequences and fewer divergent pairs.

**Table 3:** Comparison of predicted protein translations to RefSeq protein sequences.

Classification	UMD2	UMD3
Identical	5,307 (59.8%)	5,645 (65.2%)
Near-identical	1,870 (21.1%)	2,026 (23.4%)
Extensions	37 (0.4%)	29 (0.3%)
Truncations	387 (4.3%)	155 (1.8%)
Divergent ends	1,044 (11.8%)	629 (7.3%)
Gapped	69 (0.8%)	81 (0.9%)
Different	129 (1.5%)	82 (0.9%)
Other	24 (0.3%)	12 (0.1%)
**Total**	**8,867**	**8,659**

**Figure 4:**
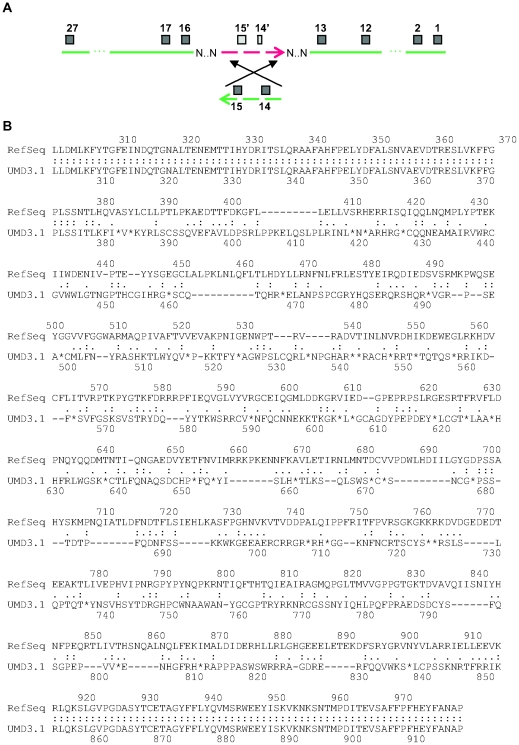
An assembly error at the *AQR* gene locus in UMD3 creates a significantly altered protein. (A) An incorrectly inverted contig (red) moves two exons (exons 14 and 15) to the wrong strand, causing the annotation software to miss them. Instead, it used low-quality alignments on the wrong strand, creating frameshifts in the predicted protein sequence that contained multiple premature stop codons. (B) Sequence alignment of the predicted AQR protein (conceptual translation) and its RefSeq model.

One example illustrating how assembly errors affect annotation is the AQR protein in UMD3. The 1422-aa sequence differs significantly from its 1484-aa RefSeq model (NP_001091560) between amino acids 380 and 851, where frameshifts introduce multiple stop codons. The reason for the discrepancy is an incorrectly oriented contig in the AQR gene region of UMD3, containing exons 14 and 15 (**Figure 4**). In place of these two exons, the annotated model of AQR seems to have used an alignment to other sequences with weak similarity to exons 14 and 15, resulting in the frameshifts. This example illustrates how protein sequence integrity reflects the quality of the assembly, and as a side benefit, it illustrates how to correct certain assembly errors.

### SNP annotation comparison

To preserve the efforts with collecting and cataloging sequence variation in a species, SNPs and other types of variations must be mapped unambiguously when a genome sequence is being replaced by a newer version. Artifacts in the assembly can interfere with SNP recovery by altering the site's sequence (genome rearrangements), by producing multiple matches (genome duplications) or by failing to identify a correct chromosomal location (unplaced sequence).

We analyzed and compared SNP annotations in the two assemblies to find the extent to which assembly quality affects the ability to store SNP information loss-free. For this purpose, SNP annotations in the two assemblies were produced by mapping the flanking sequences of ∼2.2 million cattle SNP variants in dbSNP, as described in **Methods**. The vast majority of flanking sequences for the polymorphisms reported in dbSNP represent genomic sequences that were extracted directly from assemblies utilized in their discovery (only 1,299 SNPs are annotated as being derived from cDNA). Therefore, a *de novo* SNP mapping process provides a useful metric for consistency between assemblies for the same species. Most of the SNPs could be mapped to both genomes using the search parameters, with 146,186 SNPs found only on UMD3, and a much smaller number, 4,371, found only on UMD2. Most of the assembly-specific SNPs, 100,284 of the SNPs unique to UMD3, could be mapped on the unplaced contig sequences in UMD2, which were not included in the searched genome. This result highlights the advantage of UMD3, which has significantly less unplaced sequence than UMD2.

Approximately 2.08 million SNPs (94%) could be placed in exactly one location on the UMD3 assembly, compared to only 1.88 million (85%) on UMD2. The difference of ∼200,000 mapped sequences is significant and shows that UMD3 is a better substrate for storing and representing SNPs by having more sequence placed on chromosomes and less duplicated sequence, primarily due to its better resolution of haplotype variant sequences.

## Discussion

More than 7,500 genome sequencing projects are ongoing or have already been completed [15], and the variety of represented species is staggering. As the sequencing instrument market is diversifying and new assembly algorithms are being developed, future genome projects will have to sort through a variety of options for sequencing and assembly strategies [16]. In the past, large projects to sequence model organisms have benefitted from considerable resources to produce a complete or near-complete genome sequence (http://www.genome.gov/10002154). More recently, almost all new genome projects have adopted second-generation sequencing technologies, which are far faster and cheaper but which generate shorter reads and correspondingly more fragmented assemblies (e.g., [1], [2]). The choices each project makes about read type, sequencing depth, and assembly method will affect the completeness, contiguity, and base-pair accuracy of the resulting genome sequence. By far the most widely-used byproduct of a genome sequencing project is its annotation, particularly for protein-coding genes. An important question that has not been adequately addressed, and that we attempt to address in this study, is: how much does assembly quality affect gene annotation, and how much will improvements in an assembly improve the accuracy of its genes?

Protein coding genes represent only 1–3% of a eukaryotic genome, so it is tempting to assume that errors in an assembly will have little effect on proteins. In fact, as our analyses show, inaccuracies in a genome assembly affect a large number of genes, sometimes dramatically. Local sequence errors can alter proximal sequence signals used by gene finders to predict genes, or can introduce frameshifting mutations, while contig inversions and translocations can lead to segmented and incomplete gene models. Our analysis shows that even for assemblies that have undergone extensive post-processing to improve the sequence, the consequences of assembly errors remain significant, with hundreds of genes left fragmented or incomplete. The effects are amplified in proteins, where we found that 12–20% of the sequences in both assemblies are significantly different from their corresponding RefSeq models. Duplicated regions and unplaced sequence in an assembly can hamper the efforts to recover SNPs, with 6–15% of SNPs not uniquely mappable and therefore unrecoverable in the two assemblies.

Further, improvements in the assembly are directly reflected in the quality of the annotation. Such improvements are apparent even for high-quality assemblies such as UMD2 and UMD3. Indeed, a smaller proportion of the better assembly's genes (2.8%, or 599 genes, for UMD3 versus 3.1%, or 727 genes, for UMD2) are incomplete and/or fragmented when mapped to the other assembly, due to longer contigs and scaffolds, which lead to better continuity. Similarly, more of the protein sequences (89.0% versus 81.3%) were similar to their models in UMD3 compared to UMD2, and there were fewer pairs that were divergent (8.2% versus 13.3%) or incomplete (2.7% versus 5.1%). Lastly, UMD3 was a better substrate for storing and representing SNPs, with 94% of SNPs mapped unambiguously on UMD3 versus only 85% on UMD2, owing to having more sequence placed on chromosomes and less duplicated sequence. These findings highlight the need to continue improving assemblies until a genome is truly finished.

One consequence of the practice of releasing and later improving draft genomes is that gene annotation must be updated with every release. A practical question is when to perform incremental updates versus *de novo* re-annotation. The strategy employed by many genome projects has been to track genes between assembly releases, making incremental updates. While this strategy preserves curation efforts and maintains a more consistent picture of the gene content, it is nevertheless prone to perpetuating errors and biases from earlier assemblies. When either the genome or the available annotation resources have changed substantially, *de novo* re-annotation may be more accurate, despite the added difficulties in tracking genes. In our analysis of two successive *Bos taurus* assemblies, created less than one year apart, relatively large changes in the assembly itself led to significant changes in the gene content. However, changes in the gene evidence explained two-thirds of the cases where genes could not be directly tracked between assemblies. The choice of annotation strategy, therefore, should consider both the extent of assembly changes and the amount of new evidence (e.g., new RNA-seq data sets [17]) for the species being annotated.

Lastly, the problem of annotation evaluation and comparison is itself difficult, with as many solutions as there have been studies. Although some groups have developed techniques to capture differences between tracked annotations [18], there is no standard method to compare two genomes and their annotations comprehensively and objectively. As an ever-greater number of species are captured with whole genome sequencing, the scientific community needs systematic methods to measure the accuracy of genome assembly and annotation and to compare them effectively.

## Materials and Methods

### Sequences and Gene and SNP annotations

Two versions of the *Bos taurus* genome produced at the University of Maryland [13], releases UMD2 (December 2008) and UMD3 (August 2009), were downloaded from the University of Maryland web site (http://www.cbcb.umd.edu/research/bos_taurus_assembly.shtml).

Gene annotations for the two assemblies were produced at NCBI. The NCBI eukaryotic gene prediction pipeline combines alignments of RefSeq mRNAs with gene models predicted with the program Gnomon (http://www.ncbi.nlm.nih.gov/genome/guide/gnomon.shtml), which in turn uses homology information together with *ab initio* Hidden Markov Model (HMM) algorithms to generate models of protein-coding genes. To produce the annotations, cow mRNAs, ESTs and proteins as well as other known eukaryotic protein sequences available at the time (April 2009 for UMD2 and February 2010 for UMD3) in the RefSeq database [17] were mapped to each of the two assemblies, and their partial alignments were merged to form basic gene models. These partial gene models were later used as restraints to the *ab initio* gene finding algorithm, which extended them without modifying the homology-supported portion of the gene. RefSeq mRNA alignments were chosen over the predicted models wherever available. Therefore, each transcript in the final annotation was at least partially supported by experimental evidence. Additionally, known human non-coding RNAs [18] were mapped to each genome to produce non-coding gene models. While the two snapshots of RefSeq, taken almost a year apart, may themselves cause differences between the annotations and thus complicate the comparisons, the availability of two production-quality annotations generated with the same method is uniquely valuable as a comparison tool. It also provides a realistic scenario for the differences in annotations a user can expect to see when assembly and annotation improvements come at large intervals of time, often marked by significant changes in the underlying resources. Overall, the annotation process above produced 24,901 transcripts for UMD2 (23,221 protein-coding), and 22,761 for UMD3 (21,364 protein-coding). (Gene annotations are available from our web site, above.) Of these, 8,090 loci in UMD2 and 16,040 in UMD3 were assigned names based on homology with known genes, while the rest were unnamed (or hypothetical) genes. Protein sequences were corrected to remove frameshifts before publication in GenBank, but conceptual protein translations, produced directly from the genomic annotation, were used for our analyses. Our focus was on protein-coding genes, for which annotation methods are generally more mature and reliable. Additionally, we used named and coding genes and proteins to validate the analyses and provide examples.

To annotate SNPs in the two assemblies, context sequences of cow dbSNP [19] sequences, consisting of the SNP and available flanking genomic sequence, usually 250 bp of on each side, were mapped to each genome (chromosomes 1–29 and X only) with a two-stage process. First, the fast high-throughput mapping system BLAT [20] was used to find sequences that align at high similarity, followed by a Blast [21] search to retrieve additional, lower similarity sequences. Alignments were filtered at 90% sequence identity and 90% coverage of the context sequence (‘found’ SNPs). Of those, only SNPs mapping to a unique location in the reference genome (UMD2 or UMD3) and that matched the genome at greater than 95% sequence identity over 90% or more of the length of the context sequence were considered in the comparative analysis (‘mapped’ SNPs).

### Spliced alignment

Transcript sequences from one assembly were mapped to the other assembly using ESTmapper [22], retaining all alignments longer than 100 bp with 95% or higher sequence identity. ESTmapper maps large cDNA sequence data sets to a target genome in two stages: for each query sequence, first it determines candidate regions on the genome based on shared 20-mers, and then it applies an optimized version of the sim4 algorithm [23] to align each query cDNA to each genomic regions. GMAP [24] was used to complement and validate the alignment set. Two sets of spliced alignments were constructed: the full alignment set and a subset of primary alignments, consisting of the best match only for each transcript (*n.b.*, if identical, multiple best alignments per transcript were included). The alignments were used to determine the presence or absence of a transcript in the other genome, and to find corresponding transcripts in the other annotation set.

### Transcript structure comparison

Spliced alignments of one transcript set (UMD2) were compared with the annotation on the other genome (UMD3) to determine compatible exon-intron structures and transcript correspondences between the two annotation sets. (Software for comparing gene structures is available free of charge from our web site at ftp://ftp.cbcb.umd.edu/pub/software/gencomp/.) Two exon-intron structures were compared if they overlapped by at least 50 bp and, when both transcripts had multiple exons, if they shared at least one intron. A transcript pair was deemed *compatible* if the exon-intron structures were identical along the common subinterval (a 20-bp margin of error was allowed at the ends of the exons and introns). Otherwise, the pair was said to have complex structural differences or rearrangements. Each pair of transcripts was analyzed to identify extensions, truncations or complex rearrangements. To determine a best match for each transcript in the other genome's annotation, we assigned each candidate match a priority code in the order: identical gene name and transcript evidence; identical exon-intron structures; single end extensions (other end is identical); double end extensions; single end truncations (other end is identical); double end truncation or truncation coupled with extension; and complex structural differences. If multiple candidates with the same priority code existed for a transcript, ties were broken by the largest number of common exons. As a final validation step, unique genes in one annotation were searched against the other annotation using Blast and retaining only alignments with 95% or higher sequence identity.

### Protein comparison

For each protein in the UMD2 and UMD3 annotations, we aligned the conceptual translation with the RefSeq model using Fasta [25]. Protein RefSeq models were downloaded from NCBI on 10 May 2010. Only records containing reviewed RefSeq entries (NM accessions) that were in common to the two annotations were retained; this produced 8,619 RefSeq proteins, which were used in the annotation of 8,867 genes in UMD2, and 8,659 genes in UMD3. Aligned proteins were then classified as follows: *near-identical* pairs had end-to-end alignments with few differences (≥90% sequence identity and <10 gaps); *extensions* and *truncations* had high sequence similarity (defined as above) and unaligned overhangs longer than 10 aa in one sequence only; pairs were *divergent* if the ends of both sequences could not be aligned; *gapped* alignments, resulting from the inclusion or exclusion of exons or portions of exons, had ≥90% ‘modified’ sequence identity (defined as average sequence identity of the aligned residues only, excluding gaps) with few other differences; lastly, sequences were considered *different* if they had significant amino acid differences as reflected by low percent sequence identity.
